# Potential for organ donation after controlled circulatory death: a retrospective analysis

**DOI:** 10.1097/j.pbj.0000000000000259

**Published:** 2024-07-11

**Authors:** Francisco Santos Dias, Diana Martins Fernandes, António Cardoso-Fernandes, Adriana Silva, Carla Basílio, Nuno Gatta, Roberto Roncon-Albuquerque, José Artur Paiva

**Affiliations:** a Department of Intensive Care Medicine, Centro Hospitalar Universitário de São João, Porto, Portugal; b Department of Surgery and Physiology, Faculdade de Medicina da Universidade do Porto, Porto, Portugal; c Department of Community Medicine, Information and Health Decision Sciences (MEDCIDS), Faculdade de Medicina da Universidade do Porto, Porto, Portugal; d Center for Research in Health Technology and Services, Rede de Investigação em Saúde (CINTESIS@RISE), Faculdade de Medicina da Universidade do Porto, Porto, Portugal; e Department of Internal Medicine, Hospital de Santa Luzia, Unidade Local de Saúde do Alto Minho, Viana do Castelo, Portugal; f Department of Intensive Care Medicine, Centro Hospitalar Tâmega e Sousa, Penafiel, Portugal; g Department of Medicine, Faculdade de Medicina da Universidade do Porto, Porto, Portugal

**Keywords:** kidney transplantation, liver transplantation, organ procurement, chronic kidney disease, intensive care medicine, treatment withdrawal

## Abstract

**Objectives:**

Despite the discrepancy between demand and availability of organs for transplantation, controlled circulatory death donation has not been implemented in Portugal. This study aimed to estimate the potential increase in organ donation from implementing such a program.

**Material and Methods:**

All deceased patients within the intensive care medicine department at Centro Hospitalar Universitário de São João, throughout the year 2019, were subjected to retrospective analysis. Potential gain was estimated comparing the results with the number of donors and organs collected during the same period at this hospital center. Differences in variables between groups were assessed using *t* tests for independent samples or Mann–Whitney *U* tests for continuous variables, and chi-squared tests were used for categorical variables.

**Results:**

During 2019, 152 deaths occurred after withdrawal of life-sustaining therapies, 10 of which would have been potentially eligible for donation after controlled circulatory death. We can anticipate a potential increase of 10 prospective donors, a maximum 21% growth in yearly transplantation activity, with a greater impact on kidney transplantation. For most patients, the time between withdrawal of organ support and death surpassed 120 minutes, an outcome explained by variations in withdrawal of life-sustaining measures and insufficient clinical records, underestimating the potential for controlled circulatory arrest donation.

**Conclusion:**

This study effectively highlights public health benefits of controlled circulatory arrest donation. Legislation allowing donation through this method represents a social gain and enables patients who will never meet brain death criteria to donate organs as part of the end-of-life process in intensive care medicine, within a framework of complete ethical alignment.

## Introduction

Organ donation currently encompasses living and deceased donors, the latter either after brain or circulatory death. Although in the inception of organ donation, the first donors were exclusively donors after circulatory death, since the Harvard report defining brain death in 1968,^1^ organ donation has been almost predominantly based on donation after brain death, a direct result of the relatively trouble-free medical management of the brain-dead donor, allowing for a controlled organ retrieval with minimal threat to organ viability. However, despite the potential difficulties of the donation after circulatory death, there are numerous studies demonstrating similar long-term outcomes of transplants from organs obtained through donation after brain and circulatory death.^2-5^

Between 2017 and 2019, there was an annual increase in 161 transplants in Europe, which represents a stall in organ transplantation activity.^6^ The decrease in mortality relevant to organ donation and the development of intensive care medicine, namely neurocritical care, led to a decrease in the number of brain-dead donors. The scarcity of organs available for transplantation increases the mortality of patients on the waiting list, with relevant costs for health care systems worldwide.^7^ The disparity between the supply and demand of organs available for transplantation has led several countries to reinstitute the practice of organ donation after controlled circulatory death, in the context of withdrawal of life-sustaining therapies, with a significant increase in organ supply from deceased donors.^8^

Currently, organ donation in Portugal encompasses living and deceased organ donors, in the context of brain death and uncontrolled circulatory death,^9,10^ as defined in category II of the modified Maastricht classification (Table 1). However, and in parallel with the rest of the world, the number of organ donors and the resulting organ transplantations in Portugal fall short of the needs of its population. In 2021, there were a total of 710 organ transplantations, with 3043 patients on active waiting list, 66 of whom died while waiting for an organ to become available: 25 awaiting a kidney transplant, 13 awaiting a liver transplantation, 7 awaiting a heart transplantation, and 11 awaiting a lung transplantation.^11^

**Table 1:** Modified Maastricht classification of donors after circulatory death.

Category I Uncontrolled	Found dead IA. Out-of-hospital IB: In-hospital	Sudden unexpected cardiac arrest without any attempt of resuscitation by a life-medical team
Category II Uncontrolled	Witnessed cardiac arrest IIA. Out-of-hospital IIB. In-hospital	Sudden unexpected irreversible cardiac arrest with unsuccessful resuscitation
Category III Controlled	Withdrawal of life-sustaining therapy	Planned withdrawal of life-sustaining therapy—expected cardiac arrest
Category IV Uncontrolled controlled	Cardiac arrest while brain dead	Sudden cardiac arrest after brain death diagnosis during donor life management but before planned organ retrieval

Between 2008 and 2016, the contribution of the donation after controlled circulatory death to the total number of organ transplantations was approximately 23.7% in Belgium, 49.1% in the Netherlands, and 39.1% in the United Kingdom, thereby proving its relevance in the context of organ transplantation nationwide.^8^

In our institution (Centro Hospitalar e Universitário de São João), donation after uncontrolled circulatory death was implemented in 2016, contributing in 2022 with 42% of all organ donors. Despite this, there are no available data in Portugal regarding the potential of donation after controlled circulatory death, as defined in category III of the modified Maastricht classification, as well as the possible increase in the number of organ transplantations after its implementation.

In this study, we perform a retrospective analysis of all deceased patients in the intensive care medicine department of our institution in 2019, with the aim of determining the number of potential organ donors after controlled circulatory death and the number of resulting organ transplantations, thereby estimating the impact that the implementation of a legislated, social, and ethically sound protocol aiming at the inclusion of such donors would have in Portugal.

Accordingly, the objectives of this study were to i) estimate the number of patients whose death occurred in the intensive care medicine department of our institution who would be fit for organ donation according to category III of the modified Maastricht classification^12^, ii) estimate the increase in organ transplantation resulting from the legal inclusion of these patients in the donor pool, and iii) understand the clinical context and direct causes of death in these patients.

## Materials and methods

Ethical approval for this study (Ethical Committee N° NAC 128/2023) was provided by the Ethical Committee of Centro Hospitalar Universitário de São João, Porto, Portugal, on March 24, 2023. This study was conducted with the concern and assurance of privacy and confidentiality of data in all its phases. It does not require informed consent of the participants, in accordance with Portuguese law (nº2 Clinical Research Law—Law nº 21/2014 16th April)—it is an observational clinical study with no intervention, with significant public interest, and including only deceased patients. The data collected for the purpose of this study pertain to all patients whose death occurred in the context of withdrawal of life-sustaining treatment during 2019. Patients whose death occurred unexpectedly while on active treatment were excluded from this analysis. From the collected data, we identified all patients who would be suitable for organ donation under category III of the modified Maastricht classification,^12^ according to the following inclusion criteria: younger than 75 years; absence of active neoplasia or high infection risk; absence of clinical, analytical, or imagiological findings consistent with significant compromise of kidney or liver function; and an agonal phase shorter than 120 minutes (defined as the time interval between the withdrawal of life-sustaining therapies and the patient death). These patients were defined as donors. Patients whose data were collected but did not meet these criteria were defined as nondonors. The moment of withdrawal of life-sustaining therapies was defined as the moment when the decision to shift treatment goals (from curative to comfort) was actualized, reflected in practice in the withdrawal of organ replacement or support therapies (most commonly mechanical ventilation, inotropic and vasopressor drugs, renal replacement therapies, extracorporeal circulation, etc). The diagnosis on admission to our department follows the nomenclature defined by the *International Classification of Diseases, Ninth Revision, Clinical Modification*.^13^ The potential increase in organ donation was calculated using the total number of organ donors and organ transplants in our institution during the same year. To allow for an estimation for each individual organ, the following maximum durations of the agonal phase were used: 30 minutes for liver and pancreas transplantation, 60 minutes for lung transplantation and 120 minutes for kidney transplantation. All patients whose duration of the agonal phase was not clearly discriminated were assumed to have had a duration longer than 120 minutes and, therefore, excluded from the donor group. Continuous variables were described using means and standard deviations if normally distributed, or otherwise using medians and interquartile ranges. Absolute and relative frequencies were used for categorical variables. Differences in sociodemographic and clinical variables between “donors” and “nondonors” were assessed using the independent-sample *t* test or Mann–Whitney *U* test for continuous variables. The chi-squared test was used for categorical variables. Analyses were performed using IBM SPSS Statistics 27 (IBM Corp, Armonk, New York), with 0.05 as the level of significance.

## Results

During 2019, 311 deaths occurred in patients admitted to the intensive care medicine department at Hospital de São João, 152 of which occurred after withdrawal of life-sustaining therapies and whose data were collected for the purpose of this study. The remaining 150 patients were excluded from this study because their deaths occurred unexpectedly under active treatment despite all therapeutic efforts. The baseline characteristics of the patients whose data were collected are summarized in Table 2. The sample includes 50 female patients (32.9%) and 102 male patients (67.1%). The average age at the time of death was 68 years. Cardiovascular comorbidities were present in 84.9% of all patients while respiratory comorbidities were found in 40.8%. Regarding disease severity and risk stratification scores at admission, the average *Simplified Acute Physiology Score II* was 57 (±30) and the *Acute Physiology and Chronic Health Evaluation II* score was 27 (±9.8). The average length of stay in ICU was 4 days (IQR 7.3). Organ replacement therapy was needed in 88.2% of all patients (n=134), invasive mechanical ventilation being the most frequent (73.7%). Inotropic and vasopressor support was used in 48% of all patients.

**Table 2:** Baseline and clinical characteristics comparison of patients admitted in our intensive care unit during 2019 whose death occurred by withdrawal of life-sustaining therapies according to the length of the agonal phase (shorter and longer than 120 minutes).

Sample characteristics	All patients	Donors	Nondonors	*P*
	n=152	n=10	n=142	
Demographic characteristics				0.066*
Age, median (IQR)	68 (18.5)	63.5 (10.5)	69 (19.0)	
Female, n (%)	50 (32.9)	1 (10.0)	69 (19.0)	
SAPS II, median (IQR)	57 (30)	48 (38.5)	57 (30)	0.104*
APACHE II, median (IQR)	27 (9.8)	32 (17.5)	26 (9.0)	0.091*
ICU length of stay, average-$\bar{x}$ (IQR)	4 (7.3)	3 (6.3)	4 (7)	0.586*
Time in days from admission to withdrawal decision, median (IQR)	1.0 (1.0)	3.5 (6.3)	1.0 (0.0)	0.019*
Agonal phase				0.001†
<60 min, n (%)	21 (13.8)	4 (40)	17 (11.9)	
60–120 min, n (%)	22 (14.5)	6 (60)	16 (11.3)	
>120 min, n (%)	109 (71.7)	0 (0.0)	109 (76.8)	
Organ support				
Invasive mechanical ventilation, n (%)	101 (66.4)	10 (100)	91 (64.1)	0.017†
Inotropic/vasopressor support, n (%)	60 (40.1)	1 (10.0)	58 (40.8)	0.741†
Extracorporeal membrane oxygenation, n (%)	13 (8.6)	0 (0)	13 (9.2)	1.0†

*Agonal phase*—time in minutes between the withdrawal of life-sustaining therapies and patient circulatory death defined by Portuguese legislation.^10^

* Mann–Whitney test.

† Fisher exact test.

APACHE, Acute Physiology and Chronic Health Evaluation II; IQR, interquartile range; SAPS II, Simplified Acute Physiology Score II.

In the subgroup analysis, 10 patients (6.6%) were identified as donors because they met the inclusion criteria defined, therefore being potential donors for controlled organ donation after circulatory death. The remaining 142 patients did not meet the inclusion criteria for organ donation after circulatory death, with an agonal phase longer than 120 minutes being the reason for exclusion in the majority of these patients (71.7%). In the donor's subgroup, diagnoses on admission were cardiac arrest (n=5), spontaneous cerebral hemorrhage (n=2), basilar artery occlusion (n=1), ST elevation myocardial infarction (n=1), and traumatic brain injury (n=1). In this subgroup, death after withdrawal of life-sustaining therapy occurred within 30 minutes in 30% of patients, 60 minutes in 10% of patients, and 120 minutes in 60% of patients. Sociodemographic, clinical, analytical, and imagiological characteristics of this subgroup are summarized in Table 3. The average interval between ICU admission and the decision for withdrawal of life-sustaining therapy was 3.5 days (±6.3) in the donor group and 1 day (±0) in the nondonor group (*P*=0.019, Mann–Whitney test). The average length of stay was similar in both groups. In the donor subgroup, all patients were under invasive mechanical ventilation at the time of withdrawal, unlike the nondonor subgroup (*P*=0.017, Fisher test). There were no statistically significant differences between subgroups regarding age, comorbidities, vasopressor/inotropic support, fraction of inspired oxygen, and pH value on the last available blood gas analysis before withdrawal of life-sustaining therapies. Imaging studies performed before death revealed findings compatible with chronic liver disease in one patient and multiple bilateral kidney cysts in another patient, both of whom were, therefore, excluded from potential liver and kidney donation, respectively.

**Table 3:** Sociodemographic, clinical, analytical, and imagiological characteristics of patients who would be considered organ donors after circulatory death under category III of the modified Maastricht classification (referred throughout the text as the donor group).

Sociodemographic, clinical, analytical, and imagiological characteristics	Donors (n=10)
Female, n (%)	1 (10.0)
Age, median (IQR)	63.5 (10.5)
Cardiovascular risk factors, n (%)	
Arterial hypertension	4 (40.0)
Type 2 diabetes mellitus	3 (30.0)
Dyslipidemia	3 (30.0)
Smoking habits	3 (30.0)
Obesity	2 (20.0)
Alcohol abuse	1 (10.0)
Comorbidities, n (%)	
Heart failure	1 (10.0)
Chronic kidney disease	0 (0)
Ischemic heart disease	1 (10.0)
Chronic obstructive pulmonary disease	1 (10.0)
Peripheral arterial disease	1 (10.0)
Intravenous drug abuse	1 (10.0)
Atrial flutter	1 (10.0)
Intensive care unit type, n (%)	
Polyvalent	8 (80.0)
Neurocritical	2 (20.0)
Intensive care unit length of stay, days, median (IQR)	3 (6.3)
Intensive care unit acquired infection, n (%)	
Respiratory infection	3 (30.0)
Primary bacteremia	1 (10.0)
Septic shock, n (%)	0 (0)
Patients with organ failure requiring artificial organ support, n (%)	8 (80.0)
Hemogram	
Hemoglobin (g/dL), median (IQR)	11.2 (4.5)
Platelets (/mm^3^), median (IQR)	210000 (73250)
Chemistry	
AST/TGO (U/L), median (IQR)	67 (113)
ALT/TGP (U/L), median (IQR)	46 (225.8)
Total bilirubin (mg/dL), median (IQR)	0.67 (0.19)
Urea (mg/dL), median (IQR)	37.0 (14.3)
Creatinine (mg/dL), median (IQR)	0.82 (0.34)
Sodium (mEq/L), median (IQR)	146 (7.5)
Potassium (mEq/L), median (IQR)	3.8 (0.7)
Calcium (mEq/L) median (IQR)	4.1 (0.5)
Cardiac enzymes	
High-sensitivity troponin I (ng/L), median (IQR)	739.4 (1555.7)
Myoglobin (ng/mL), median (IQR)	188.1 (273.8)
Arterial blood gas	
pH, median (IQR)	7.40 (0.08)
pO2 (mmHg), median (IQR)	96.6 (54.3)
pCO2 (mmHg), median (IQR)	40.5 (4.8)
Lactate (mmol/L), median (IQR)	1.4 (0.7)
PaO2/FiO2 class, n (%)	
<100	1 (10.0)
100–200	2 (20.0)
200–300	4 (40.0)
>300	3 (30.0)
Imaging	
Liver imaging, n(%)	
Imagiological test suggesting chronic liver disease with or without cirrhosis	1 (10.0)
Imagiological test suggesting hepatic steatosis	1 (10.0)
Imagiological test suggesting primary or secondary neoplastic liver masses	0 (0)
No relevant findings on liver imaging	3 (30.0)
No available liver imaging	5 (50.0)
Kidney imaging, n (%)	
Kidney imaging suggesting chronic kidney disease	0 (0)
Imagiological test suggesting primary or secondary neoplastic kidney masses	1 (10)
No relevant findings on kidney imaging	6 (60)
No available kidney imaging	3 (30)

Considering the maximum duration of the agonal phase defined in this study and organ imaging examinations performed before death, the inclusion of these donors in the donor pool would lead to an additional 2 lung, liver, pancreas, and kidney donors; 1 lung, pancreas, and kidney donor; 1 lung and kidney donors; and 5 kidney donors. Hence, the overall number of organs available estimated are 2 livers, 3 pancreas, 8 lungs, and 18 kidneys (Table 4). The most commonly accepted definition of expanded criteria for kidney donation is any dead donor older than 60 years or 50 years with two of the following: a history of hypertension, the most recent serum creatinine greater than or equal to 1.5 mg/dL, or death resulting from a cerebral vascular accident.^14^ This definition arose from several analyses of the US scientific registry of transplant recipients, which defined expanded kidney donation according to the donor characteristics that corresponded to an increase in 70% of the risk of graft failure or loss.^15-17^ Our institutional guidelines differ only in the age range, defining expanded criteria donors as those aged 65 years or older. According to this definition, 4 of the 10 potential donors met criteria for expanded donation because 3 of them were older than 65 years and another patient had a history of hypertension, factors that could influence decisions regarding organ donation in these patients. Of note, however, there is international variation of this definition, with the example of the Eurotransplant Senior Program, which defines older donors as those aged 65 years or older and allocates these organs to patients of the same age range.^18^ In the same time frame, 47 deceased donors were registered in our institution.^19^ Ultimately, a program for organ donation under controlled circulatory death would lead to an increase in 21% of all yearly transplant activities in our institution.

**Table 4:** Total number of potentially transplantable organs from patients who would be considered organ donors after circulatory death under category III of the modified Maastricht classification.

	Number of organ donors	Number of transplantable organs
Liver (agonal phase <30 minutes)	2	2
Pancreas (agonal phase <30 minutes)	3	3
Lung (agonal phase <60 minutes)	4	8
Kidney (agonal phase <120 minutes)	9	18

*Agonal phase—*time in minutes between the withdrawal of life-sustaining therapies and patient’s circulatory death defined by Portuguese legislation.^10^

## Discussion

The study demonstrates a potential 21% annual growth in transplantation activity with the implementation of a cDCD program, at Centro Hospitalar Universitário de São João. The estimated gain surpasses the outlined objectives described in the literature, which advocate for a combined 10% increase in transplantation activity across the European Union over a decade, an already ambitious goal.^6^ Notably, the most significant contribution would occur in renal transplantation, consistent with results from other countries using cDCD protocols.^8^ The data suggest an anticipated increase of 10 deceased kidney donors, a significant rise compared with the total of 78 renal transplants performed at the same center in 2019.^19^

Classically, the time limits for the agonal phase after which donation must be abandoned are 30 minutes for the liver and pancreas, 60 for the lung, and 120 for the kidney.^8^ The intervals represent an estimate of warm ischemia time. Owing to the retrospective nature of this study and lack of hemodynamic parameters specifically regarding the interval between withdrawal of life-sustaining therapies and death, it was impossible to assess warm ischemia time in each potential donor. However, the relationship between the length of the agonal phase and graft function remains controversial.^20-22^ In fact, evidence suggests that, more than the elapsed time since cessation of active treatment, the events occurring during the agonal phase are more preponderant for the prospect and viability of organ donation.^23^ Therefore, protocols exist where the decision to interrupt donation is determined by the time elapsed since the onset of functional warm ischemia (systolic blood pressure lower than 50 mmHg or oxygen saturation lower than 70%), as opposed to the time since cessation of organ support. This strategy allows the inclusion of donors who would otherwise be excluded for exceeding classical time limits.

The time between withdrawal of organ support and death exceeded 120 minutes in the majority of patients (71.1%). Typically, this period lasts an average of 20 minutes, and most cDCD donors die within 120 minutes.^24,25^ In our institution, there is no written protocol for withdrawal of life-sustaining therapies and there is some heterogeneity of attitudes, depending on the patient and physician. However, the general standard practice is to withdraw mechanical ventilation either through terminal extubation or reduction of ventilatory settings to physiological parameters, and/or to discontinue vasopressor and inotropic support. The absence of guiding standards for withdrawal of life-sustaining measures and end-of-life care poses a barrier to the development of cDCD programs.^26^ A notable proportion of patients continued to receive some form of organ support beyond the decision to withdraw active treatment, potentially extending the agonal phase. This, in turn, led to the exclusion of potential donors, underestimating the results. Terminal withdrawal of mechanical ventilation and discontinuation of inotropes, vasopressors, and extracorporeal membrane oxygenation shorten the agonal phase and warm ischemia time. A legislative change allowing cDCD would make shortening the warm ischemia time an ethically and clinically relevant goal. The second constraint is related to the insufficiency of clinical records concerning the strategy of active treatment withdrawal and the timings of decisions. Considering these limitations, patients with unclear or undocumented time intervals between the suspension of life-sustaining measures and death were excluded, potentially underestimating even further the pool of donors.

Experience and expertise with cDCD programs, including donor maintenance within these programs, have expanded in the past decade. The success of such programs is currently well established, not only for kidney transplantation but also for heart, lung, liver, and pancreas transplants.^26^ Donation through cDCD allows the procurement of multiple organs because teams can coordinate the suspension of organ support with the availability of surgical teams, a strategy that expedites the harvesting process. The results are well established even without resorting to sophisticated reperfusion strategies, which should not be a barrier to successful renal donation through cDCD but are essential in hepatic and cardiac transplantation.^26^

Heart failure and irreversible neurological disease were the main reasons for admission, consistent with international experience. Typically, cDCD includes patients with catastrophic brain injuries who do not meet criteria for brain death and patients with respiratory or heart failure.^24^ The time between admission and the decision to withdraw organ support was longer in the potential donor's subgroup, but consistent with the available literature reporting an average duration of four days.^27^ The literature suggests that this time frame helps boost clinicians' confidence in the prognosis and decision making regarding suspension of organ support. In addition, it improves the experience of families around the decision-making process and end-of-life care.^26^

Intensive care length of stay was 3 days, resembling the European average.^28^ In this regard, concerns about physical or human resources should not become barriers to cDCD programs. A donor adds an additional 30.8 years of life, distributed on average among 2.9 solid organ recipients,^29^ and represents a social gain of 7.3 quality-adjusted life years (QALY) per occupied intensive care bed per day. If all possible organs for transplantation are considered, this gain can reach 10.8 QALY. For all these reasons, increasing the potential for cDCD is a public health priority, with unquestionable social benefits.

Introducing a cDCD program requires a legal framework for the retrieval of organs for transplantation from patients whose death is diagnosed and confirmed using cardiorespiratory criteria. Such criteria are well established and socially accepted in Portugal since 2013.^11^ Under the umbrella, uDCD is a reality in Portugal, but cDCD is not yet practiced, despite the socially accepted tradition of curtailing medical treatment when futile. The decision-making process, grounded by principles of beneficence and autonomy, is a multidisciplinary one, shared with the patient or their representatives, in alignment with the values and wishes of the individual patient. cDCD programs also offer the possibility for individuals who will never meet brain death criteria to fulfil their wish for organ donation. In countries where this reality is established, there is a growing social acceptance of organ donation as part of the end-of-life process in intensive care, within a framework of total ethical alignment.

## Conclusion

There is presently a clear international discrepancy between the need and availability of organ donors, which results in long waiting periods for organ transplantation, with negative consequences for patients with end-stage chronic organ failure and increased health care costs for all health services.^29^

This study allows for an estimation in the increase of organ availability with the inclusion of controlled organ donation after circulatory death, as defined in category III of the modified Maastricht classification. In our institution, in particular, this would lead to an increase in 21% of all yearly transplant activities. These results are conservative estimations, based on the intentionally cautious premises of this study, with strict inclusion and exclusion criteria, potentially incurring in an underestimation of the potential contribution of this form of donation but with low risk of an overestimation.

The implementation of a program for controlled donation after circulatory death demands a strict legal framework and the optimization of certain aspects, such as the correct identification of potential donors, the definition of eligibility criteria, and the standardization of intervention with a clear and swift response both inside the hospital and between different health institutions. It is our duty, both individually and collectively, to ensure that all physical and human assets are available for its implementation.
